# #Black Lives Matter

**DOI:** 10.1038/s42003-020-1062-6

**Published:** 2020-06-22

## Abstract

*Communications Biology* is committed to improving diversity in our pages, our reviewer pool, and our editorial board. We recognize both the overt and unconscious racism in the sciences and have tasked ourselves with using our platform to amplify Black voices and the voices of all biologists of color.

We, the editors of *Communications Biology*, are deeply saddened by the recent events in the U.S.: the killings of George Floyd, an unarmed Black man; Breonna Taylor, a Black woman who was asleep in her own home; and countless others, by police officers. These events are of course only the tip of the iceberg and have sparked mass protests nationwide in demand of social reform and justice. The systemic and deeply rooted racism reflected in this and too many other similar events is not confined to any border or culture or field. Change is not easy and doesn’t happen overnight; however, it feels that in the last few years, the political environment in many countries has moved every inch of forward change yards backwards, and amplified the problems that had previously simmered just below the surface. So how can we as a society—and as scientists—affect positive change?

It all starts with us. Before pointing fingers at others, we must self-reflect.

As scientists, we know intellectually that racism makes no sense. All humans share nearly identical DNA, and we know that most genetic variation is found within populations rather than between them^1^. Therefore, it follows that science should be accessible to all people, regardless of group identification. Unfortunately, the reality is that we still suffer from wide racial disparities in STEM. Just look around any academic department or research lab; how many Black scientists do you find? Despite some progress in the last 10 years, in 2017 Black Americans still accounted for only 5.6% of the work force in science and engineering, while representing 11.9% of the population in the U.S^2^.

Our journal offices are based in big cities like New York City and London—cities that are often referred to as ‘melting pots’—where you would expect to have the highest levels of diversity. Still, even research institutes in these cities suffer from a dramatic under-representation of Black professionals in the sciences and in science publishing. This disparity doesn’t start at the professional level. It is shaped by inequities throughout life in terms of access to good schools and colleges, mentorship opportunities and support infrastructure. An example of this can be found in the UK, where institutional racism against Black students and employees on campuses is not seriously recognized or addressed, which pushes many of the students to seek alternatives in other countries or leave academia^3^. This raises the question: are we as publishers doing enough to encourage equal opportunity in academia and in science research more broadly? Equal opportunity isn’t achieved by posting a job advert and including the one-liner “we encourage applications from minorities”. It is likewise not achieved by passively managing manuscript submissions and inviting reviewers without making a conscious effort to improve diversity.

So what can we do?

At *Communications Biology*, we will seize on this opportunity to educate ourselves, prioritize efforts to reach Black scientists at all stages of their careers, and ensure that such efforts are part of a long-term continuous plan rather than a one-time reaction to a specific incident or to show that we are checking a box. Instead, we pledge to speak out against racism and reflect on actionable steps for a positive change in the life sciences. After careful thought, we share our commitments:

“We pledge to speak out against racism and reflect on actionable steps for a positive change in the life sciences.”

- We will work to recruit Black scientists to our editorial board.
- We will start a series to discuss the journey in science for Black researchers at any point in their career. This will be in the form of a Q&A series that sheds light on the scientific journey of Black and other minority researchers.
- We will aim for each of our in-house editors to visit at least one Historically Black College or University, or other research institute with a majority of students from underrepresented minorities, per year to talk to students about careers in science publishing, to promote editor recruitment and to provide seminars on the publication process and publishing in high-impact journals for researchers.
- We will actively seek volunteers from underrepresented groups, including Black biologists, to review manuscripts, in order to enrich our reviewer pool and ensure it reflects the full community of biologists. To achieve this, we have added a question to capture this dimension of reviewer diversity to our volunteer reviewer spreadsheet. Please do suggest your colleagues and scientist friends.
- We will highlight scientists who are actively working towards the enrichment of opportunities for Black individuals, or who are focusing their research on Black health and environmental disparities, through commissioned Comment and Perspective articles.
- We will strive to actively encourage and recruit Black editors to our in-house team and the teams of our sister journals. Please don’t hesitate to contact us about opportunities at Nature Research if this is something you may be interested in.

Racism is not a political issue, but rather a human rights issue. Science and human rights go hand-in-hand. With this in mind, we at *Communications Biology* stand in solidarity with the Black community and with our fellow activists in the Black Lives Matter movement to not only denounce racism, but to actively commit to ensuring equal opportunity and achieving antiracism. As a single journal, we cannot solve the inequities caused by hundreds of years of systemic racism. But by working together, an all-inclusive future becomes possible.
